# Supplementary figures and images for: COVID-19 infections and outcomes in a live registry of heart failure patients across an integrated health care system
Source: PLoS One. 2020 Sep 30;15(9):e0238829. doi: 10.1371/journal.pone.0238829 (PMC7526909; doi:10.1371/journal.pone.0238829)

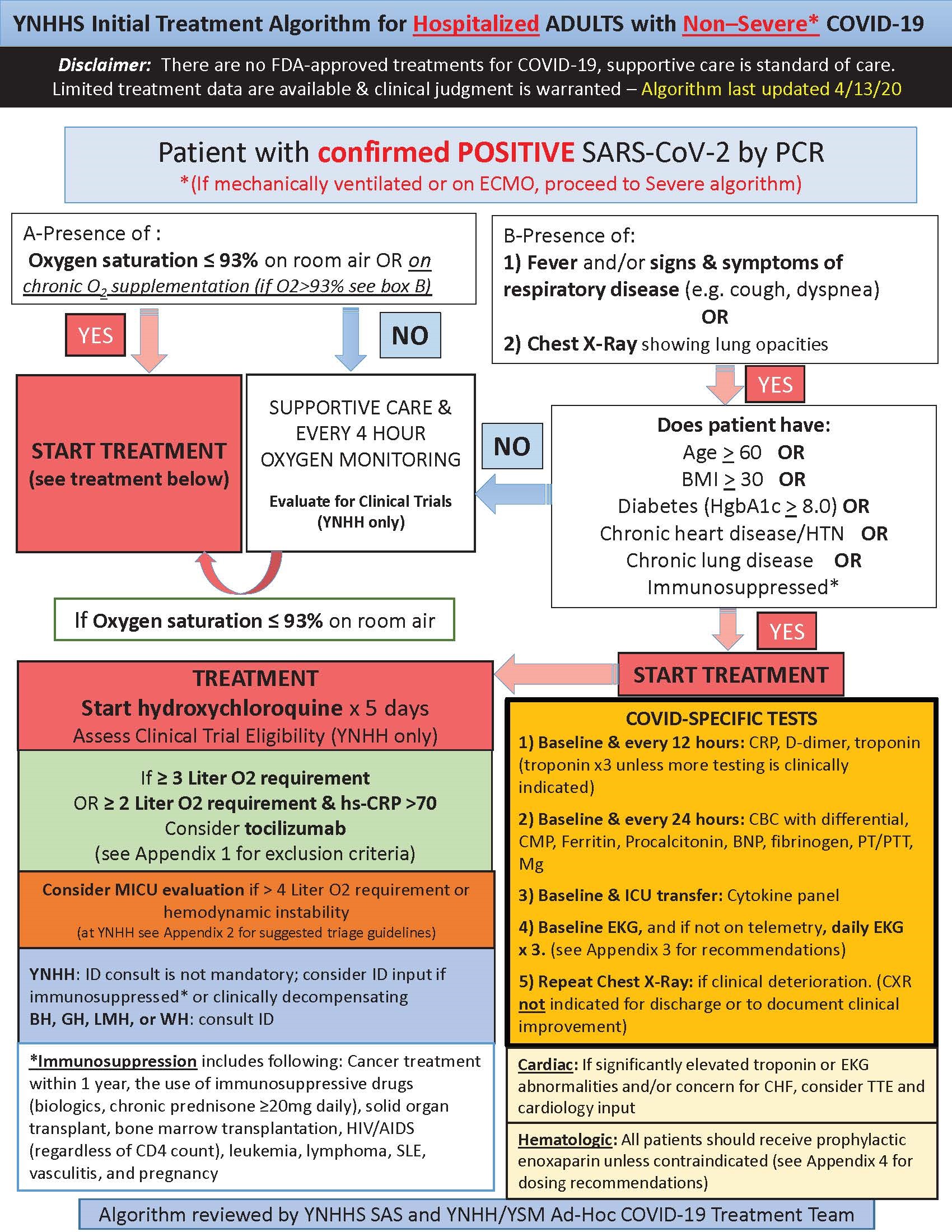

Supplement: S1 Fig — (JPG) [file pone.0238829.s001.jpg]

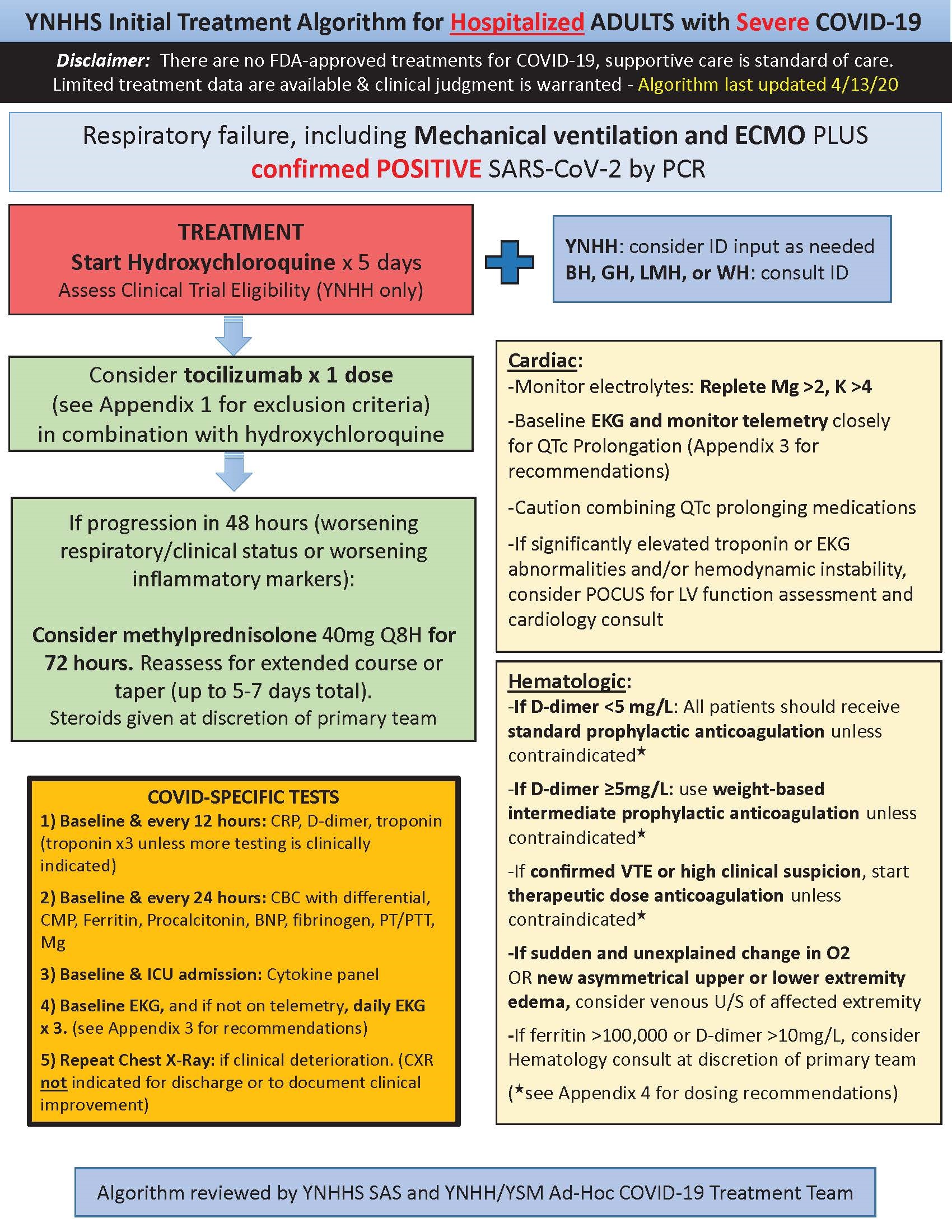

Supplement: S2 Fig — (JPG) [file pone.0238829.s002.jpg]
