# Supplementary material for: COVID-19 infections and outcomes in a live registry of heart failure patients across an integrated health care system
Source: PLoS One. 2020 Sep 30;15(9):e0238829. doi: 10.1371/journal.pone.0238829 (PMC7526909; doi:10.1371/journal.pone.0238829)
Supplement: Attachment — Submitted filename: Review Plose one.doc [file pone.0238829.s003.doc]

Caraballo C. and Collegues described the social and clinical profile, the prevalence and the predictors of outcomes of large sample of HF patients positive to SARS-CoV-2, registered in the YALE Heart Failure Registry in Connecticut area.
Being able to use a real-time registry the authors have clearly described the clinical differences of 900 symptomatic COVID+ HF patients in respect those COVID- and the predictors of poor outcomes in this subgroup of HF patients.
Although some clinical data had already been confirmed elsewhere, in this study some interesting findings must to be underline: the elevated rate of death (20%) in very elderly (>85 years) HF patients with symptomatic COVID, the negative prognostic role of high burden of comorbid conditions associated with low level of socioeconomic status. This data, together of other clinical evidences, clearly suggest how HF older frail patients should be the priority for any prevention program of vaccination or other preventive care program. Also interesting is the data concerning the positive association RAAS inhibitor drugs and prognosis; moreover this result is found in a large population of patients in whom ace-inhibitors have a high level of evidence based recommendation. Another flag finding concerns the low percentage of HF patients underwent testing for COVID, this report, in my opinion, is another important clinical alert that must be taken into account by those who have health- care system responsibilities.
Thus I think that this paper, well written, deserves a good consideration for publication in the Journal accounting the aforementioned considerations.

Obviously this is a retrospectively analysis but in my opinion it does not reduce significantly the clinical value.
